# Poor glycaemic control and ectopic fat deposition mediates the increased risk of non-alcoholic steatohepatitis in high-risk populations with type 2 diabetes: Insights from Bayesian-network modelling

**DOI:** 10.3389/fendo.2023.1063882

**Published:** 2023-02-22

**Authors:** T. Waddell, A. Namburete, P. Duckworth, A. Fichera, A. Telford, H. Thomaides-Brears, D. J. Cuthbertson, M. Brady

**Affiliations:** ^1^ Department of Engineering Science, The University of Oxford, Oxford, United Kingdom; ^2^ Perspectum Ltd, Oxford, United Kingdom; ^3^ Department of Computer Science, The University of Oxford, Oxford, United Kingdom; ^4^ Oxford Robotics Institute, The University of Oxford, Oxford, United Kingdom; ^5^ Department of Cardiovascular and Metabolic Medicine, Institute of Life Course and Medical Sciences, University of Liverpool, Liverpool, United Kingdom; ^6^ Liverpool University Hospitals NHS Foundation Trust, Liverpool, United Kingdom

**Keywords:** Ectopic fat deposition, magnetic-resonance imaging, type-2 diabetes, non-alcoholic steatohepatitis, body composition

## Abstract

**Background:**

An estimated 55.5% and 37.3% of people globally with type 2 diabetes (T2D) will have concomitant non-alcoholic fatty liver disease (NAFLD) and the more severe fibroinflammatory stage, non-alcoholic steatohepatitis (NASH). NAFLD and NASH prevalence is projected to increase exponentially over the next 20 years. Bayesian Networks (BNs) offer a powerful tool for modelling uncertainty and visualising complex systems to provide important mechanistic insight.

**Methods:**

We applied BN modelling and probabilistic reasoning to explore the probability of NASH in two extensively phenotyped clinical cohorts: 1) 211 participants with T2D pooled from the MODIFY study & UK Biobank (UKBB) online resource; and 2) 135 participants without T2D from the UKBB. MRI-derived measures of visceral (VAT), subcutaneous (SAT), skeletal muscle (SMI), liver fat (MRI-PDFF), liver fibroinflammatory change (liver cT1) and pancreatic fat (MRI-PDFF) were combined with plasma biomarkers for network construction. NASH was defined according to liver PDFF >5.6% and liver cT1 >800ms. Conditional probability queries were performed to estimate the probability of NASH after fixing the value of specific network variables.

**Results:**

In the T2D cohort we observed a stepwise increase in the probability of NASH with each obesity classification (normal weight: 13%, overweight: 23%, obese: 36%, severe obesity: 62%). In the T2D and non-T2D cohorts, elevated (*vs.* normal) VAT conferred a 20% and 1% increase in the probability of NASH, respectively, while elevated SAT caused a 7% increase in NASH risk within the T2D cohort only. In those with T2D, reducing HbA1c from the ‘high’ to ‘low’ value reduced the probability of NASH by 22%.

**Conclusion:**

Using BNs and probabilistic reasoning to study the probability of NASH, we highlighted the relative contribution of obesity, ectopic fat (VAT and liver) and glycaemic status to increased NASH risk, namely in people with T2D. Such modelling can provide insights into the efficacy and magnitude of public health and pharmacological interventions to reduce the societal burden of NASH.

## 1 Introduction

According to the World Health Organisation, the global prevalence of obesity almost tripled between 1975 and 2016 with 39% of the world’s adult population (1.9 billion; 39% of men, 40% of women) overweight and 13% (650 million; 11% of men, 15% of women) living with obesity in 2016 (1). In parallel, according to the International Diabetes Federation, the global diabetes prevalence in 2019 was estimated to be 9.3% (463 million people), with a projected 25% increase by 2030 (10.2% prevalence; 578 million) and a projected 50% increase (10.9% prevalence, 700 million) by 2045 (2).

People living with obesity and type 2 diabetes (T2D) are at a significantly greater risk of liver related complications compared to people without either condition (3). Notably, according to a recent global meta-analysis and meta-regression, ~55.5% of people with T2D worldwide have associated non-alcoholic fatty liver disease (NAFLD), 37.3% non-alcoholic steatohepatitis (NASH) and 17.3% biopsy-confirmed advanced liver fibrosis (4). Those people with NAFLD and concomitant T2D have significantly worse liver-related outcomes including higher rates of advanced fibrosis, cirrhosis and liver-related cancers compared to those with NALFD only (5–8).

20-year projections of the economic and clinical burden of NASH/NAFLD estimate that co-prevalent NASH and T2D will account for 65,000 liver transplants, 812,000 liver-related deaths and 1.37 million cardiovascular-related deaths, totalling $55.8 billion in healthcare costs (9). Early detection of those patients with T2D at high-risk of NASH is therefore of considerable importance and would enable early access to personalised care/medicines and improved clinical and disease outcomes.

In both T2D and NASH, obesity is a significant risk factor, though the clinical utility of the body mass index (BMI) metric is limited since it describes global body mass relative to height and does not describe body fat distribution. People with T2D represent a clinical population, and relative to those without T2D, are characterised by a distinct body composition profile with significantly higher volumes of visceral adipose tissue (VAT), increased liver fat deposition and fibroinflammation and reduced skeletal muscle mass (10, 11), when measured by magnetic resonance imaging (MRI). Furthermore, elevated VAT but not subcutaneous adipose tissue (SAT), has been associated with a significant increase in circulating insulin, plasma glucose and incidence of the metabolic syndrome (12, 13), highlighting the importance of studying body fat distribution.

Multi-parametric MRI can provide quantitative tissue characterisation in multiple organs. Fat infiltration (steatosis), iron deposition and fibroinflammatory change can be measured using proton density fat fraction (PDFF), iron and fat corrected T1 mapping (cT1) with MRI. PDFF quantifies liver fat at each voxel and correlates strongly with histologic steatosis (14, 15) while liver cT1, an indicator of liver disease activity and severity, correlates strongly with histological markers of fibroinflammation and demonstrates high diagnostic accuracy for stratifying patients with NASH and those with at-risk NASH (16, 17).

In this paper, we use MRI-derived measures of body composition and liver health using PDFF and liver cT1 to identify participants with NAFLD and NASH and to overcome the intrinsic limitation of the BMI noted above by exploring regional fat distribution. We describe how applying Bayesian-networks to study the associations between MRI-derived measures of body composition and liver health, enables a comprehensive assessment of the high-risk metabolic phenotypes associated with co-prevalent T2D and NASH. We also show how BNs can be used to identify potential therapeutic targets for alleviating NASH risk.

## 2 Methods

### 2.1 Data collection and preparation

We investigated two datasets: the first included 221 participants with T2D pooled from the MODIFY study (NCT04114682) (18) and the UK Biobank (UKBB) online resource under application 9914. MODIFY recruited participants with T2D from primary or secondary care settings from three sites across the UK; UKBB is a general population-based cohort study in people aged 40 to 69 years from across the UK (https://www.ukbiobank.ac.uk/). The second cohort included 135 participants without diabetes of any kind (non-T2D) drawn from the UKBB. All participants underwent an abdominal MR scan that included multi-parametric imaging of the liver and pancreas. See (10) for an in-depth description of the MR-protocol.

### 2.2 Clinical details and MRI image acquisition and analysis

**Figure 1:**
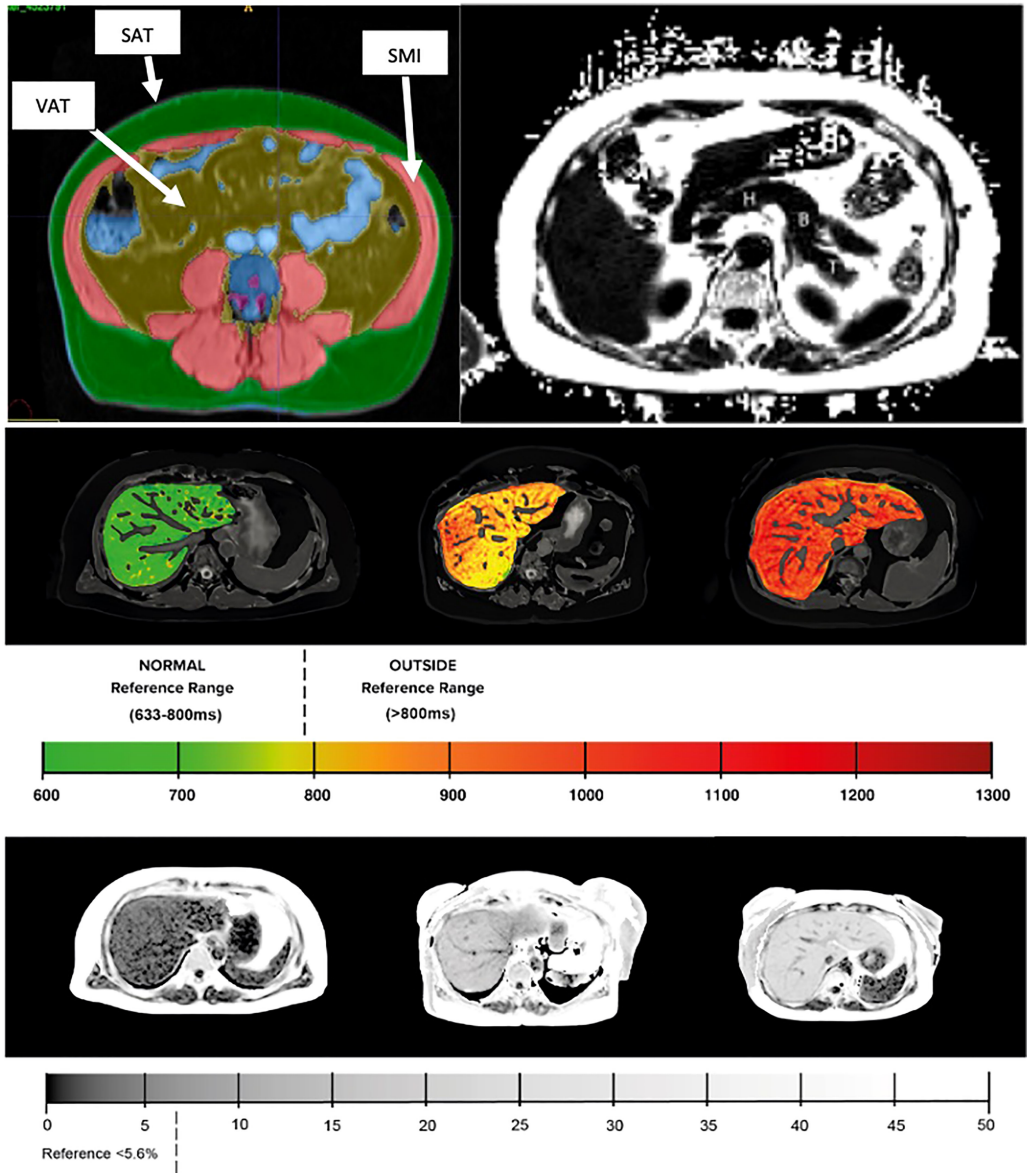
(Top left) Example MR images of body composition segmentation: SMI, skeletal muscle index; SAT, subcutaneous adipose tissue; VAT, visceral adipose tissue. (Top right) pancreas with example typical ROI placement (H – head, B – body, T – tail). (Middle) liver cT1 and (bottom) liver PDFF with corresponding reference values.

Body composition was examined from a 2D MR slice positioned at the third lumbar (L3) vertebra and measurements of VAT, SAT and skeletal muscle were based on manual delineations by trained analysts, see **Figure 1**. Measures of skeletal muscle were then indexed to the participant’s height to produce a measure of skeletal muscle index (SMI) (cm^2^/m^2^). All continuous variables were discretized based on pre-defined clinical thresholds or by splitting the data into ‘low’ and ‘high’ value groups determined at the 75^th^ percentile value, see supplementary. For example, HbA1c within the T2D cohort was discretised by splitting the cohort into ‘low’ (HbA1c <62mmol/mol) and ‘high’ (HbA1c >=62mmol/mol) value groups. Presence of NASH was classified based on liver PDFF >5.6% and liver cT1 >800ms. Such thresholds have consistently demonstrated high diagnostic accuracy for stratifying patients with NASH, predicting liver-related outcomes, classifying between NASH and NAFLD only and are an effective alternative to liver biopsy for diagnosing NASH (17, 19).

*Bayesian-networks (see Supplementary information for full explanation of BNs)*

Bayesian Networks (BNs) are directed acyclic graphs that are capable of explicitly representing and analysing complex systems and under certain assumptions, specifying causal relationships. When combined with probabilistic reasoning, BNs can be used to estimate the probability of an ‘event’ occurring in response to a fixed evidence input. For example (20), used BNs to estimate the probability of acute kidney injury after fixing certain biochemical abnormalities in patients with gastrointestinal cancer.

To investigate the probability of NASH in specific clinical characteristics, we conducted conditional probability queries that estimate the probability of an event (here, NASH), given an ‘evidence list’ containing the set values of specific network variables (for example, obesity or HbA1c status). The probability of NASH was estimated using the likelihood weighting algorithm, a Monte Carlo approximation technique utilising importance sampling. While BNs can be extended to incorporate a plethora of clinical features and sources of information, our networks have been deliberately limited to illustrate their application of the present analysis.

### 2.3 Network variables

BN construction included the following variables: liver fat (PDFF %), liver cT1 (ms), pancreatic fat (PDFF %) visceral adipose tissue (cm^2^), subcutaneous adipose tissue (SAT) (cm^2^), skeletal muscle (SMI) (cm^2^), gender (1[male]/0[female]), BMI (kg/m^2^), age (yrs), HbA1c (mmol/mol), AST (IU/L), ALT (IU/L), smoking status (0[non-smoker]/1[current smoker]/2[past smoker]).

### 2.4 Bayesian-network construction (See Supplementary information for full overview of network construction)

**Figure 2:**
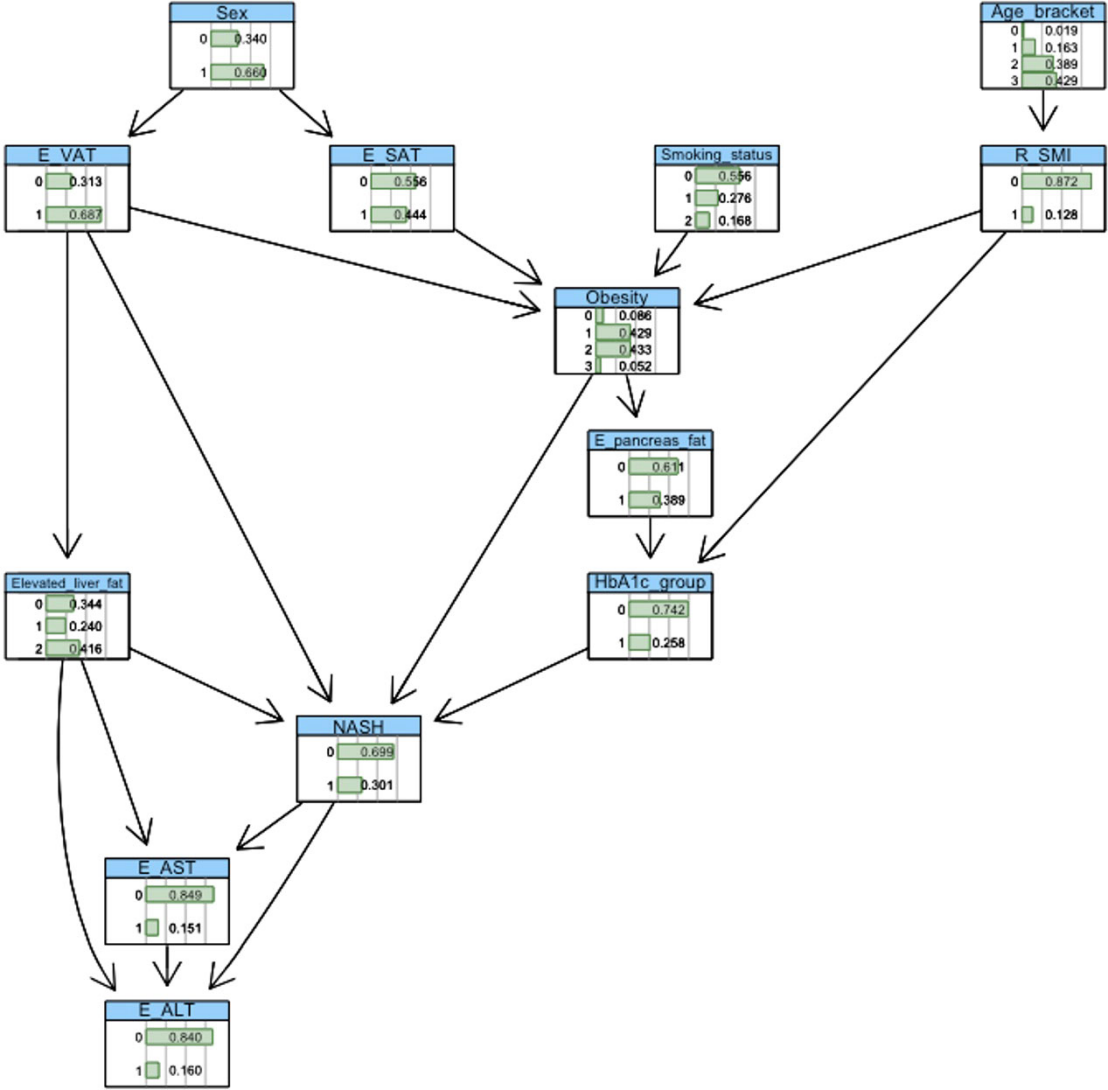
Bayesian-network structure from the T2D cohort.

**Figure 3:**
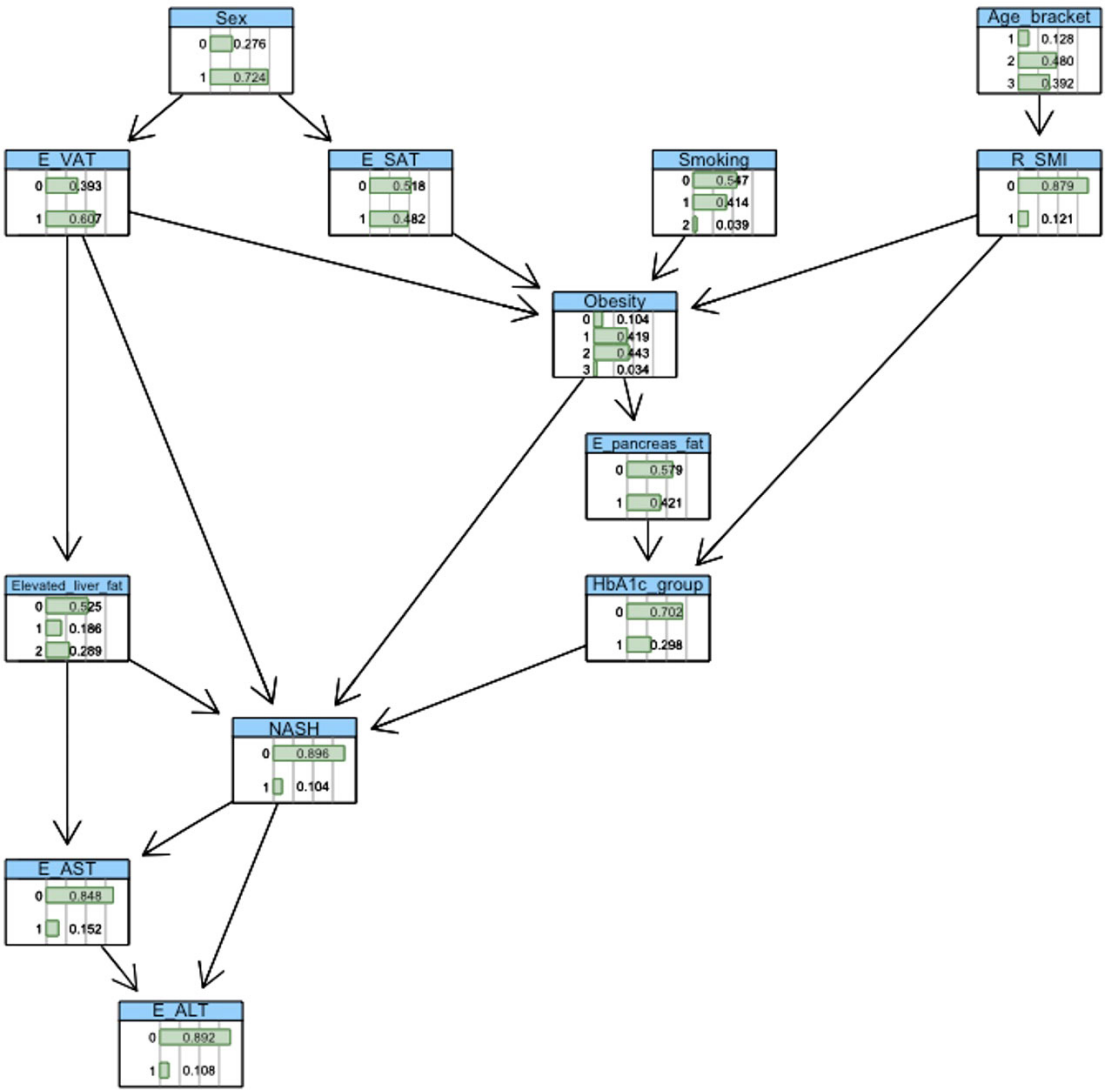
Bayesian-network structure from the non-T2D cohort.

BN construction and probabilistic inference were completed using the ‘bnlearn’ package and visualised using ‘graphviz’ within the R software platform (version 3.6.1). Automated network structures, derived from a score-and-search algorithm (21), were adjusted by removing or reversing nonsensical edges and inserting edges based on domain knowledge gleaned from medical literature. Crucially, incorporation of clinical knowledge in these network structures enables the modelling of *causal* relationships between variables. **Figures 2**, **3** show the final networks from the T2D and non-T2D cohorts, respectively.

### 2.5 Statistical analysis

All statistical analyses used the R software platform (version 3.6.1). Descriptive statistics, showing median [inter quartile range], are reported to summarise population characteristics. Wilcoxon and X^2^ tests were used to make between group comparisons.

## 3 Results

### 3.1 Descriptive characteristics of participants

The baseline clinical, biochemical, and imaging characteristics are shown in **Table 1**. The groups were well matched for age, gender, and BMI. Despite similar liver biochemistry, participants from the T2D cohort (and thus higher HbA1c (<0.001)) had significantly elevated liver cT1 (<0.001), liver PDFF (<0.001), VAT/SAT ratio (p=0.034) and greater prevalence of NASH (<0.001).

**Table 1:** Baseline characteristics of T2D and non-T2D cohorts.

Characteristic	T2D (n = 221)	Non-T2D (n = 135)	p-value
Clinical data			
Age (yrs)	57 [52-64]	57 [53-63]	0.3
Sex (n of male participants [%])	146 (66%)	98 (73%)	0.2
BMI (kg/m^2^)	30 [27-34]	29 [26-33]	0.3
HbA1c (mmol/mol)	50 [41-62]	36 [33-38]	**<0.001**
AST (IU/L)	24 [19-29]	27 [23-32]	**<0.001**
ALT (IU/L)	27 [20-36]	26 [19-35]	0.5
MRI data			
Liver cT1 (ms)	751 [695-827]	709 [672-752]	**<0.001**
Liver fat (%)	8 [4-14]	5 [3-11]	**<0.001**
Pancreatic fat (%)	5 [3-9]	5 [3-8]	0.6
VAT (cm^2^)	238 [173-307]	215 [149-279]	0.065
SAT (cm^2^)	249 [180-322]	271 [181-366]	0.3
VAT/SAT ratio	0.90 [0.58-1.33]	0.79 [0.57-1.15]	0.034
SMI (cm^2^/m^2^)	49 [42-56]	51 [43-56]	0.6
NASH (n of participants [%])	69 (31%)	13 (9%)	**<0.001**

Data is median [IQR]. Bold denotes significance.

### 3.2 Estimation of NASH using probabilistic reasoning

The baseline probabilities of NASH in the T2D and non-T2D cohorts were 30% and 10%, respectively. ‘Intervening’ on a variable within the BN means that a specific value is assigned to it. For example, the variable ‘HbA1c’ may be assigned the values ‘0’ (‘low’ value) or ‘1’ (‘high’ value). The effect of amending level of glucose regulation is then propagated throughout the network, where the variables(s) conditionally dependent on the intervened variable(s) are updated to reflect the specified evidence.

### 3.3 T2D cohort

#### 3.3.1 Impact of body mass index

**Figure 4:**
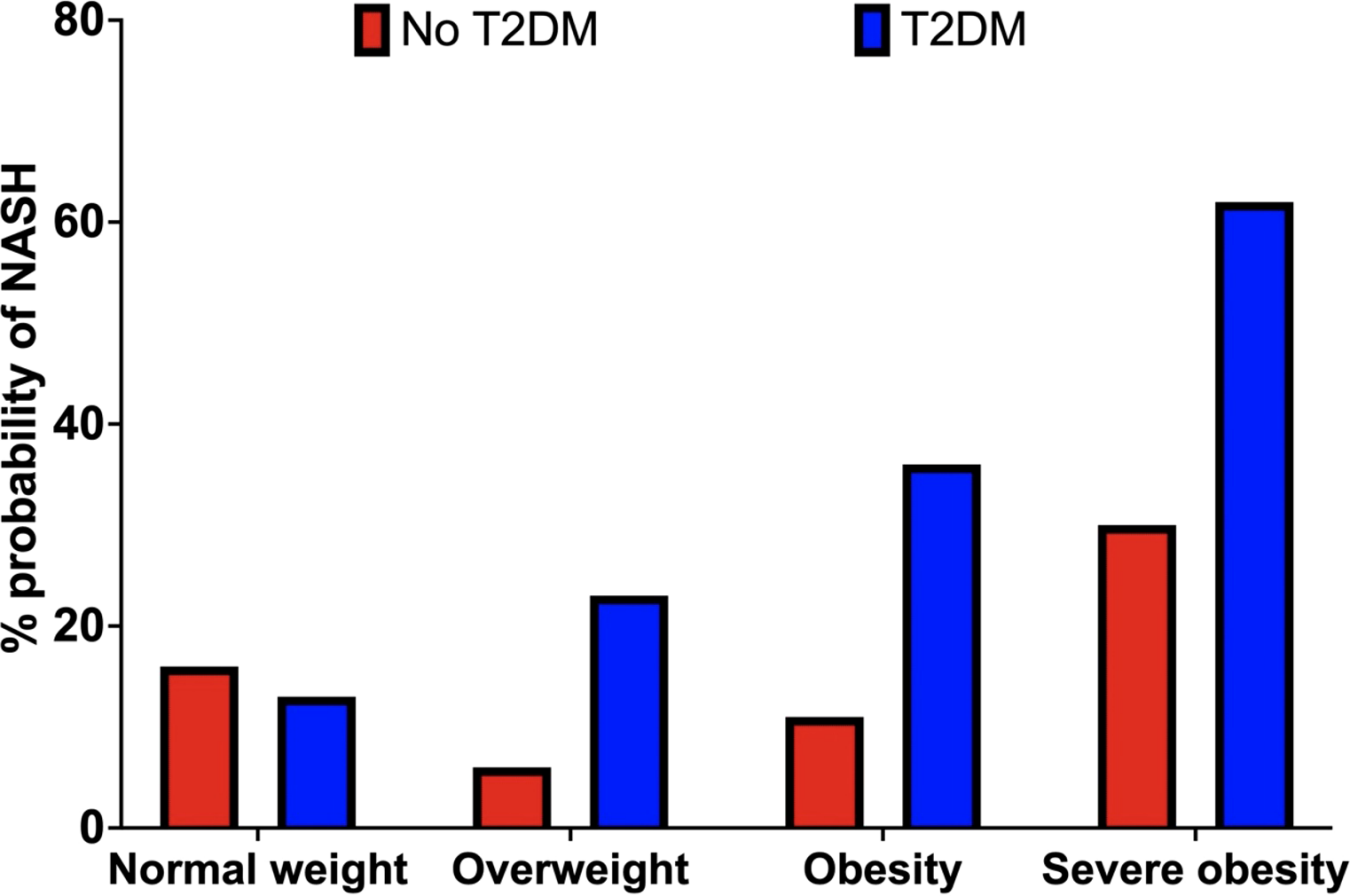
Probability of NASH (%) by obesity category (BMI) in the non-T2D (red) and T2D (blue) cohorts.

We first explored the effect of obesity status on NASH risk, observing a 13%, 23%, 36% and 62% probability of NASH under normal weight (<25kg/m^2^), overweight (25-30kg/m^2^), obesity (30-40kg/m^2^), and severe obesity (>40kg/m^2^) settings, respectively. This equated to a 23% greater probability of NASH when comparing ‘obesity’ *vs.* ‘normal weight’. (**Table 2**; **Figure 4**).

**Table 2:** Probability of NASH (%) under different variable settings in the T2D and non-T2D cohorts.

Biomarker	Cohort	
	T2D (n = 221)	Non-T2D (n = 135)
Obesity status (BMI)		
Normal weight (<25kg/m^2^)	13%	16%
Overweight (25-30kg/m^2^)	23%	6%
Obese (30-40kg/m^2^)	36%	11%
Severe obesity (>40kg/m^2^)	62%	30%
Body composition & fat deposition		
Normal VAT	16%	10%
Elevated VAT	36%	11%
Normal SAT	27%	10%
Elevated SAT	34%	10%
Normal SMI	30%	10%
Reduced SMI	26%	11%
Liver fat 5.6-10%	25%	7%
Liver fat >10%	56%	30%
Normal pancreatic fat	29%	10%
Elevated pancreatic fat	30%	10%
HbA1c range		
Low (<62mmol/mol [T2D]; <38mmol/mol [non-T2D])	86%	30%
High (>=62mmol/mol [MODIFY]; >=38mml/mol [non-T2D])	67%	27%

#### 3.3.2 Impact of HbA1c

**Figure 5:**
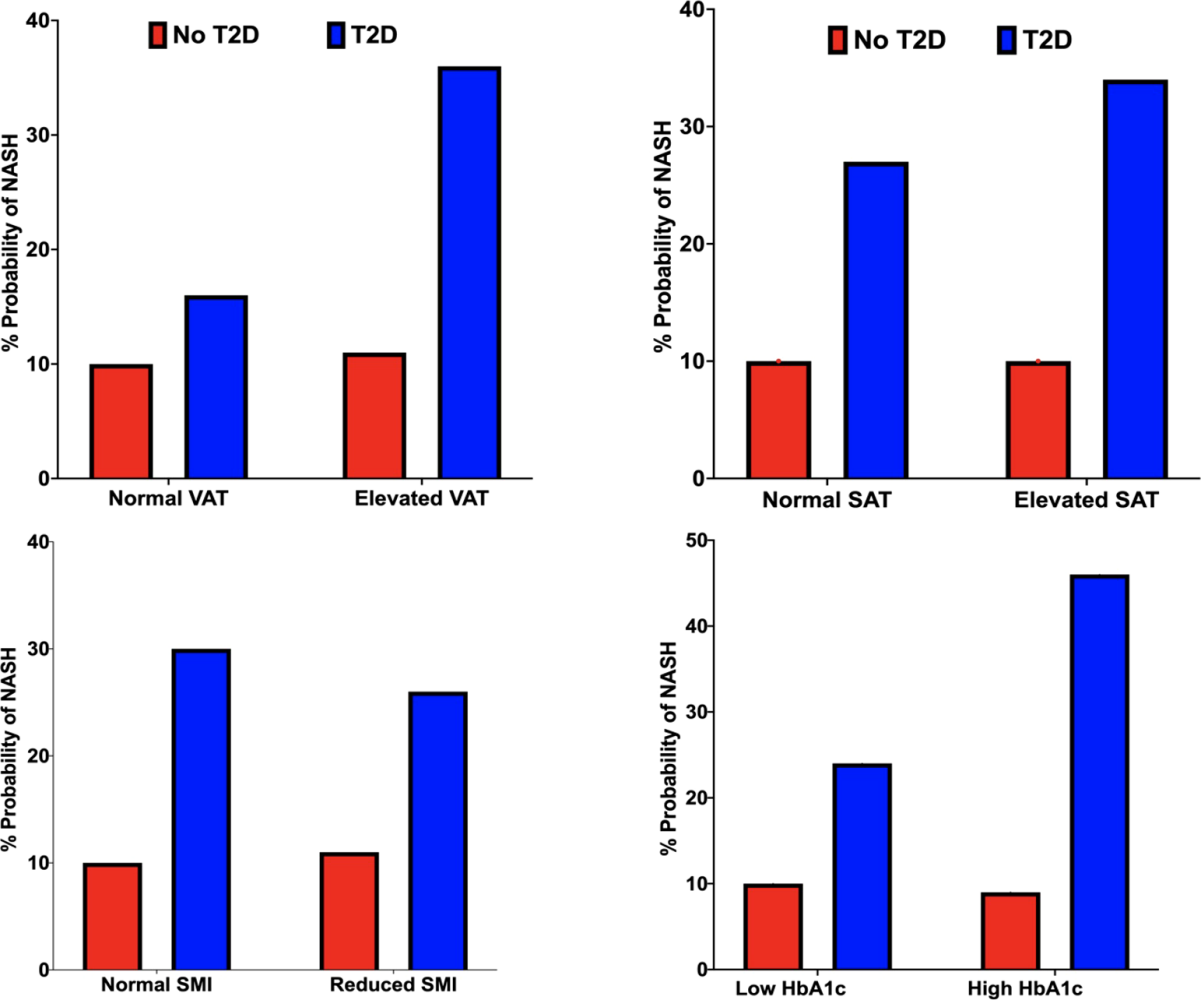
Probability of NASH (%) under VAT (top left), SAT (top right), SMI (bottom left) and HbA1c status (bottom right) conditions in the non-T2D (red) and T2D (blue) cohorts.

Increasing HbA1c status from the low (HbA1c <62 mmol/mol) to high value group (HbA1c >=62mmol/mol) increased the probability of NASH by 22% (24% vs. 46%). (**Table 2**; **Figure 5**).

#### 3.3.3 Impact of adipose tissue volumes (VAT and SAT)

We observed a 20% and 7% increase in the probability of NASH when specifying elevated vs. normal measures of VAT and SAT, respectively. (**Table 2**; **Figure 5**).

*Impact of skeletal muscle area:* Reduced SMI decreased the probability of NASH by 4% (30% vs 26%). (**Table 2**; **Figure 5**).

#### 3.3.4 Impact of liver and pancreatic fat

Increasing liver fat from 5.6-10% to greater than 10% increased the probability of NASH by 31% (25% vs 56%). Elevated pancreatic fat increased the probability of NASH by 1% (29% vs 30%). (**Table 2**; **Figure 5**).

#### 3.3.5 Combination of risk factors

BNs enable a user (e.g., clinician) to specify an individual phenotype, enabling personalised ‘what if’ analysis. For example, a high-risk phenotype with obesity, elevated VAT, elevated liver fat (>10%) and ‘high’ HbA1c had an 86% probability of NASH. In this phenotype, reducing HbA1c to the ‘low’ value reduced the probability of NASH by 29% (67%).

### 3.4 Cohort without T2D

We observed a 16%, 6%, 11% and 30% probability of NASH under normal weight, overweight, obese, and severe obesity settings. We observed a 1% increase in the probability of NASH when specifying elevated vs normal measures of VAT. Increasing liver fat from 5.6-10% to >10% increased the probability of NASH by 23% (7% vs. 30%). Increasing HbA1c from the ‘low’ <38mmol/mol) to ‘high’ (>38mmol/mol) value reduced the probability of NASH by 1% (10% vs 9%). (**Table 2**; **Figures 4**; **5**).

Exploring the same high-risk phenotype specified within the T2D cohort, we observed a 30% probability of NASH. Reducing HbA1c lowered the probability of NASH by 3% (27%).

## 4 Discussion

In this paper, we applied a novel BN approach to demonstrate the mediating influence of type 2 diabetes, particularly determining the impact of poor glycaemic control and higher ectopic fat deposition, focussing on the impact of liver and visceral fat depots, on the increased risk of NASH in people with T2D and those in the general population. Firstly, we found that obesity *(versus* normal BMI) conferred a 23% increase (within the T2D cohort) and 5% decrease (in the cohort without T2D) in the probability of NASH. However, considering the intrinsic limitations of the BMI, aggregating all measures of body composition (for example, combining adipose tissue and skeletal muscle volumes) into a single measure, this metric assumes that all individuals will have a similar relative proportion of the different biological tissues.

Our work overcomes this limitation by applying BNs to study the association between MR-derived measures of body composition (using regional adipose tissue volumes, VAT and SAT and organ specific fat measurements) and NASH risk, highlighting the independent contribution of fat deposition within visceral and subcutaneous sites. Specifically, within the T2D cohort the probability increase of NASH was more than double under elevated VAT (+20%) than elevated SAT (+7%) conditions. Such finding is similar to that of (22) who, despite applying different statistical techniques, also observed that higher measures of VAT relative to SAT was predictive of advanced liver fibrosis in people with NAFLD.

VAT area is independently associated with NASH and correlates significantly with histology confirmed NAFLD with significant fibrosis (23). Proposed mechanisms behind the association of elevated VAT and NASH include lipotoxicity and an overexpression of proinflammatory cytokines that promote inflammation and fibrosis within the hepatocytes (24). Such overexpression of cytokines has been linked to fat deposition within visceral, but not subcutaneous, adipose sites (25).

In participants with T2D, our analysis revealed a 22% reduction in the probability of NASH when lowering HbA1c from the ‘high’ (>62mmol/mol) to ‘low’ (<62mmol/mol) range. Furthermore, NASH risk was reduced by 29% when lowering HbA1c in an example high risk phenotype. Importantly, this was achieved without specifying changes to obesity status, liver fat content or body composition. Chronically elevated blood glucose (i.e., glucotoxicity) has been linked to NASH development *via* its effects on increasing TCA cycle activity and synthesis of Acyl CoA that promote *de novo* lipogenesis and oxidative stress (26). At the time of writing, no FDA-approved pharmacological medications are available for the treatment of NASH. To this end, our findings highlight the potential of glucose lowering therapies for mitigating NASH risk, particularly in high-risk metabolically unhealthy populations or those with overweight/obesity. Interestingly, newer T2D therapies such as GLP1-receptor agonists (liraglutide, semaglutide), novel dual and triple peptides (e.g. tirzepatide) and SGLT2-inhibitors have been shown to decrease levels of ALT and lower liver fat (measured by MRI-PDFF) in people with T2D and areas of fibrosis, ALT/AST and hepatic lipid content in murine NASH models (27, 28).

Notably, and despite similar measures of BMI and body composition (see **Table 1**), we found significantly greater measures of liver cT1, liver fat and prevalence of NASH within the T2D cohort. This highlights the clinical need to screen people with T2D for concomitant liver disease and NASH, where critically, we observed similar measures of ALT and significantly greater AST in the non-T2D cohort. Screening for liver disease needs to adopt a multi-modality approach that extends beyond the reliance on circulating biomarkers alone.

Limitations of this study ultimately derive from the fact that NASH is a complex and multifactorial disease that involves numerous mechanisms that are not investigated in our work to date. However, the BN methodology that we use is intrinsically extensible. Future work will seek to broaden the variables included in network construction, such as biochemical pathways associated with hepatocyte injury, for a more comprehensive assessment of NASH risk.

In conclusion, this paper applied Bayesian-networks and probabilistic reasoning to identify populations at a high risk of NASH. We emphasise the role of elevated VAT, liver fat and obesity status in driving the probability of NASH, and these effects are most significant in people with type 2 diabetes highlighting the importance of prevention and good glycaemic management as a potential therapeutic target for addressing the epidemic of NAFLD/NASH.

## Data availability statement

The data analyzed in this study is subject to the following licenses/restrictions: Private data. UK Biobank restrictions. Requests to access these datasets should be directed to tom.waddell@perspectum.com.

## Author contributions

TW analyzed the data, built the Bayesian-network, conducted the probabilistic reasoning statistics, and wrote the manuscript. PD and AN supported in revising the manuscript and offered technical assistance in study methodology. HT-B supported in revising the manuscript. DC provided clinical support in building the network, devising probabilistic reasoning questions, revising the manuscript, and producing figure plots. AT provided technical assistance on statistical methodology. AF collected and synthesized genetic data from the UK Biobank. MB assisted with drafting the manuscript, defining project scope, and revising the manuscript for publication. All authors contributed to the article and approved the submitted version.
