# Supplementary material for: Poor glycaemic control and ectopic fat deposition mediates the increased risk of non-alcoholic steatohepatitis in high-risk populations with type 2 diabetes: Insights from Bayesian-network modelling
Source: Front Endocrinol (Lausanne). 2023 Feb 22;14:1063882. doi: 10.3389/fendo.2023.1063882 (PMC9992174; doi:10.3389/fendo.2023.1063882)
Supplement: Supplementary file 1 [file DataSheet_1.docx]

***Methodology of Bayesian networks***

Bayesian-networks (BN) are composed of 1) a directed acyclic graph (DAG) specifying conditional dependencies between network variables, and 2) a set of conditional probability distributions attached to each variable within the DAG. Formally, a DAG is expressed as G = (V, E), where V = {X_1_, X_2_, …, X_n_} denotes random variables within the network and E ⊆ {V x V} a set of directed edges connecting V. Due to their acyclic nature, under certain assumptions BNs can provide a convenient visual representation of causal relationships.

Concretely, a variable - $x_{1}$- is conditionally independent of its non-descendants *given* its parents within the network, denoted $P\left( x_{1} \right|{Pa (x}_{1}))$, with the value of $x_{1}$ conditionally dependent on that of its parent ($Pa)$ node(s). Such conditional dependencies are factorised to form a joint probability distribution across the entire network via the chain rule:

$P \left( x_{1}, x_{2}, \ldots, x_{n} \right)=P {(x}_{1}) P \left( x_{2} \right|x_{1})\ldots P \left( x_{n} | x_{1}x_{2}\ldots x_{n}-_{1} \right)$ (1)

Such factorisation also allows a compact representation of the joint probability distribution of a specific variable, for $x_{1}$ and $x_{2}$ will not appear in each other’s conditioning set if deemed conditionally independent given G.

An example BN is shown in **Figure 1**, where the edges from ‘X’ and ‘Z’ to ‘Y’ denote the direction of *causality*. Such edges explicitly model the conditional dependencies between variables, where in the given example, the value of ‘Y’ is conditionally dependent on the values of ‘X’ and ‘Z’. Though a potentially powerful tool, special care needs to be taken when applying BNs to study *causality* in healthcare, for the presence and orientation of edges need to be consistent with medical knowledge.

**Supplementary Figure 1.** Simplified example Bayesian-network. Here, the value of ‘Y’ is conditionally dependent on the values of ‘X’ and ‘Z’.

***Bayesian-network construction***

The score-based Hill-Climbing structure learning algorithm [1] with Bayesian Information Criterion (BIC) provided the initial network construction, see **Figure 2**. These networks were then adjusted by removing or reversing nonsensical edges, such as the edge from ‘elevated SAT’ to ‘Sex’, and inserting edges based on domain knowledge gleaned from medical literature, such as the edge from ‘elevated liver fat’ to ‘NASH’. Crucially, incorporation of clinical knowledge in these network structures enables the modelling of *causal* relationships between variables, for the presence and direction of edges are not simply bias dependencies within the dataset. Variable parameters were fitted using the ‘Bayes’ method with uniform priors within the bn.fit function.


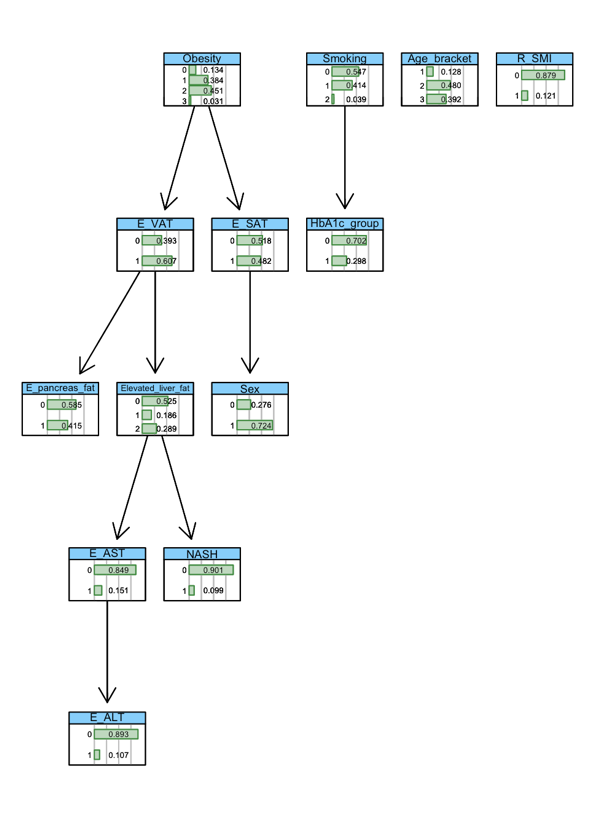

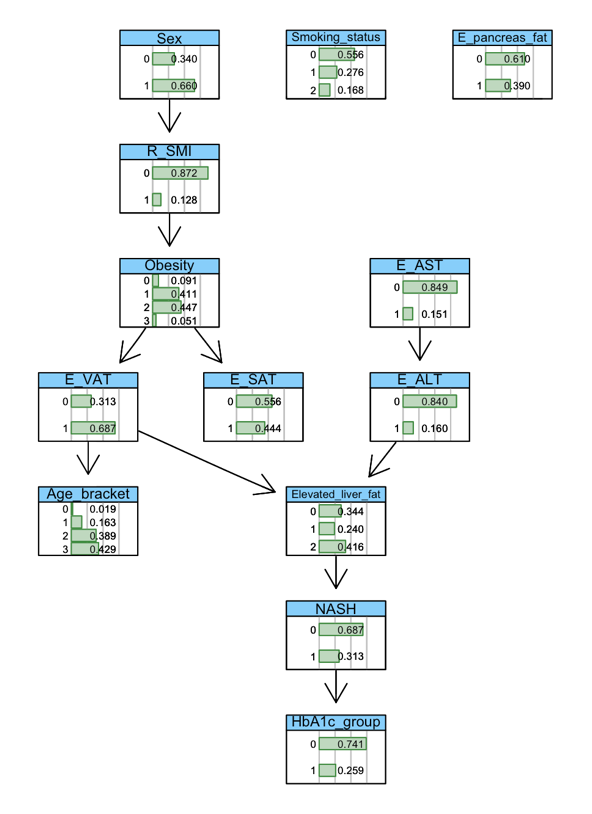


**Supplementary Figure 2.** Automated network structures derived from the T2D (left) and non-T2D (right) cohorts.

**References**

1. Selman, B. and Gomes, C.P., 2006. Hill-climbing search. Encyclopedia of cognitive science, 81, p.82.
