# Supplementary material for: Poor glycaemic control and ectopic fat deposition mediates the increased risk of non-alcoholic steatohepatitis in high-risk populations with type 2 diabetes: Insights from Bayesian-network modelling
Source: Front Endocrinol (Lausanne). 2023 Feb 22;14:1063882. doi: 10.3389/fendo.2023.1063882 (PMC9992174; doi:10.3389/fendo.2023.1063882)
Supplement: Supplementary file 2 [file Table_1.docx]

**Supplementary Table 1.** Discretisation thresholds.

| **Variable** | **Discretisation** |
| --- | --- |
| Liver PDFF (%) | [0] - <5.6%  [1] – 5.6-10%  [2] - >10% |
| Liver cT1 (ms) | [0] - <800ms  [1] – 800-875ms  [3] - >875ms |
| Pancreatic PDFF (%) | [0] - <6.6%  [1] - >6.6% |
| VAT (cm^2^) | **Male**  [0] - <210(cm^2^)  [1] - >210(cm^2^)  **Female**  [0] - <140(cm^2^)  [1] - >140(cm^2^) |
| SAT (cm^2^) | **Male**  [0] - <220(cm^2^)  [1] - >220(cm^2^)  **Female**  [0] - <400(cm^2^)  [1] - >400(cm^2^) |
| SMI (cm^2^/m) | **Male**  [0] - >44(cm^2^/m)  [1] - <44(cm^2^/m)  **Female**  [0] - >31(cm^2^/m)  [1] - <31(cm^2^/m) |
| Body mass index (kg/m^2^) | [0] - <25(kg/m^2^)  [1] – 25-30 (kg/m^2^)  [2] – 30-40(kg/m^2^)  [3] - >40(kg/m^2^) |
| Age (yrs) | [0] - <40yrs  [1] – 40-50yrs  [2] – 50-60yrs  [3] – 60-70yrs  [4] - >70yrs |
| HbA1c (mmol/mol) | **T2D**  [0] - <62mmol/mol  [1] - >62mmol/mol  **Non-T2D**  [0] - <38mmol/mol  [1] - >38mmol/mol |
